# A population in perpetual motion: Highly dynamic roosting behavior of a tropical island endemic bat

**DOI:** 10.1002/ece3.9814

**Published:** 2023-02-11

**Authors:** Samantha Aguillon, Gildas Le Minter, Camille Lebarbenchon, Axel O. G. Hoarau, Céline Toty, Léa Joffrin, Riana V. Ramanantsalama, Stéphane Augros, Pablo Tortosa, Patrick Mavingui, Muriel Dietrich

**Affiliations:** ^1^ UMR PIMIT (Processus Infectieux en Milieu Insulaire Tropical) Université de la Réunion/INSERM1187/CNRS9192/IRD249 Sainte‐Clotilde France; ^2^ Cabinet ECO‐MED Océan Indien Saint‐Denis France

**Keywords:** Chiroptera, Molossidae, reproductive seasonality, Reunion Island, sexual segregation, tropical island bat

## Abstract

Although island endemic bats are a source of considerable conservation concerns, their biology remains poorly known. Here, we studied the phenology and roosting behavior of a tropical island endemic species: the Reunion free‐tailed bat (*Mormopterus francoismoutoui*). This widespread and abundant species occupies various natural and anthropogenic environments such as caves and buildings. We set up fine‐scale monitoring of 19 roosts over 27 months in Reunion Island and analyzed roost size and composition, sexual and age‐associated segregation of individuals, as well as the reproductive phenology and body condition of individuals. Based on extensive data collected from 6721 individuals, we revealed a highly dynamic roosting behavior, with marked seasonal sex‐ratio variation, linked to distinct patterns of sexual aggregation among roosts. Despite the widespread presence of pregnant females all over the island, parturition was localized in a few roosts, and flying juveniles dispersed rapidly toward all studied roosts. Our data also suggested a 7‐month delay between mating and pregnancy, highlighting a likely long interruption of the reproductive cycle in this tropical bat. Altogether, our results suggest a complex social organization in the Reunion free‐tailed bat, with important sex‐specific seasonal and spatial movements, including the possibility of altitudinal migration. Bat tracking and genetic studies would provide additional insights into the behavioral strategies that shape the biology of this enigmatic bat species. The fine‐scale spatiotemporal data revealed by our study will serve to the delineation of effective conservation plans, especially in the context of growing urbanization and agriculture expansion in Reunion Island.

## Graphical abstract

We studied the phenology and roosting behavior of a tropical island endemic species: *Mormopterus francoismoutoui*. We set up a fine‐scale monitoring of 19 roosts over 27 months in Reunion Island and collected extensive data from 6721 individuals. We revealed a highly dynamic roosting behavior, with marked seasonal sex‐ratio variation, linked to distinct patterns of sexual aggregation among roosts. Our study provides a relevant example of how dynamic are sex‐specific behavioral strategies in bats, even for tropical species living in small oceanic islands.

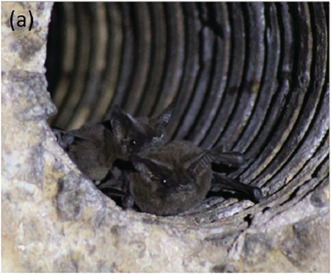

## 1 INTRODUCTION

Islands are unique but vulnerable ecosystems. Indeed, islands shelter a higher proportion of endemic taxa than do equivalent continental areas, and this diversity is disproportionally more sensitive to both natural and anthropogenic disturbance, such as cyclonic storms, urbanization, habitat degradation, or species introductions (Brooks et al., 2002; Elton, 2000; Jones et al., 2009; McCreless et al., 2016; Myers et al., 2000). Due to their ability to disperse over water, bats are often the only native mammals on islands, especially on remote oceanic islands (e.g., the Hawaiian hoary bat *Lasiurus semotus* in Hawaii; Baird et al., 2015; Jones et al., 2009; Pinzari et al., 2020). More than half of known bat species live on islands, and 25% are endemic to islands (Jones et al., 2009). Island endemic bats provide key services to insular ecosystems, including arthropod regulation, pollination, and seed dispersal (Aziz et al., 2017; Kalka & Kalko, 2006). For example, the flying fox *Pteropus niger* plays an important role in dissemination and regeneration of native plants on the oceanic island of Mauritius in the Indian Ocean (Florens et al., 2017). Endemic bats are considered as a significant conservation concern and represent around 50% of the world's threatened bats (Conenna et al., 2017; Jones et al., 2009). The ability of bat populations to recover after natural catastrophes or human perturbations depends on their size, ability to move, and social behavior (Jones et al., 2009). Therefore, knowledge of the ecology of island bats is crucial for the delineation and success of sustainable conservation projects.

The Reunion free‐tailed bat (*Mormopterus francoismoutoui*; Goodman et al., 2008) is a small insectivorous tropical bat endemic to Reunion Island, and is the most abundant bat species compared to the two others living on the island: the Mauritian flying fox (*P. niger*), a fruit bat, and the Mauritian tomb bat (*Taphozous mauritianus*), an insectivorous bat. Reunion Island is a small volcanic territory (2512 km^2^), that emerged about 3 million years ago (Cadet, 1980) in the south‐western Indian Ocean (Mascarene Archipelago), and shaped by a very steep mountainous landscape. The tropical climate of the island is recognized by two seasons: a hot rainy season and the dry season, defined here as summer and winter, respectively. Through 350 years of human colonization, Reunion Island ecosystems have suffered from deforestation, agricultural expansion, and urbanization (Lagabrielle et al., 2009). Despite this extensive landscape fragmentation, the Reunion free‐tailed bat is broadly distributed on the island, mostly in the lowland and urbanized areas (Augros et al., 2015; Barataud & Giosa, 2013; Moutou, 1982). Roosts, that are always monospecific, are home to a few hundred to tens of thousands of individuals (Augros et al., 2015; Dietrich et al., 2015). This species occupies a variety of day roosts such as caves and crevices within cliff faces, as well as anthropogenic settings such as buildings and bridges (Goodman et al., 2008). This bat follows a similar pattern to many other species of Molossidae found elsewhere in the world, that have the capacity to adapt to urban or modified habitats (Jung & Kalko, 2011).

Despite being the only exclusive endemic mammal of Reunion Island, little information is available about the biology and population dynamics of the Reunion free‐tailed bat. The size of the island population has never been precisely assessed but could reach several hundreds of thousands of individuals (Augros et al., 2015; Dietrich et al., 2015). Based on the study of the largest known maternity roost (ca. 46,500 adult individuals), Dietrich et al. (2015) confirmed that parturition occurs during austral summer, from December to February, and that the roost is empty during austral winter (June to September), suggesting seasonal movements between roosts (Goodman et al., 2008). However, the mating period remains unidentified, and social organization within and between roosts is largely unknown, as the occupancy of roosts by different types of individuals (male/female, adult/juvenile) has never been investigated. The goal of the present study was to examine the phenology and roosting behavior of the Reunion free‐tailed bat. We set up a fine‐scale monitoring of multiple roosts all over the island during a 27‐month period in order to estimate temporal variations of roost size and composition (age and sex). We evaluated temporal trends in the segregation of individuals within roosts, relative to sex and age classes, and analyzed the effect of roost size on such patterns. We also described the reproductive phenology in this tropical bat species, and measured the influence of factors shaping body condition of individuals.

## 2 MATERIALS AND METHODS

### 2.1 Study sites and sampling frequency

**FIGURE 1:**
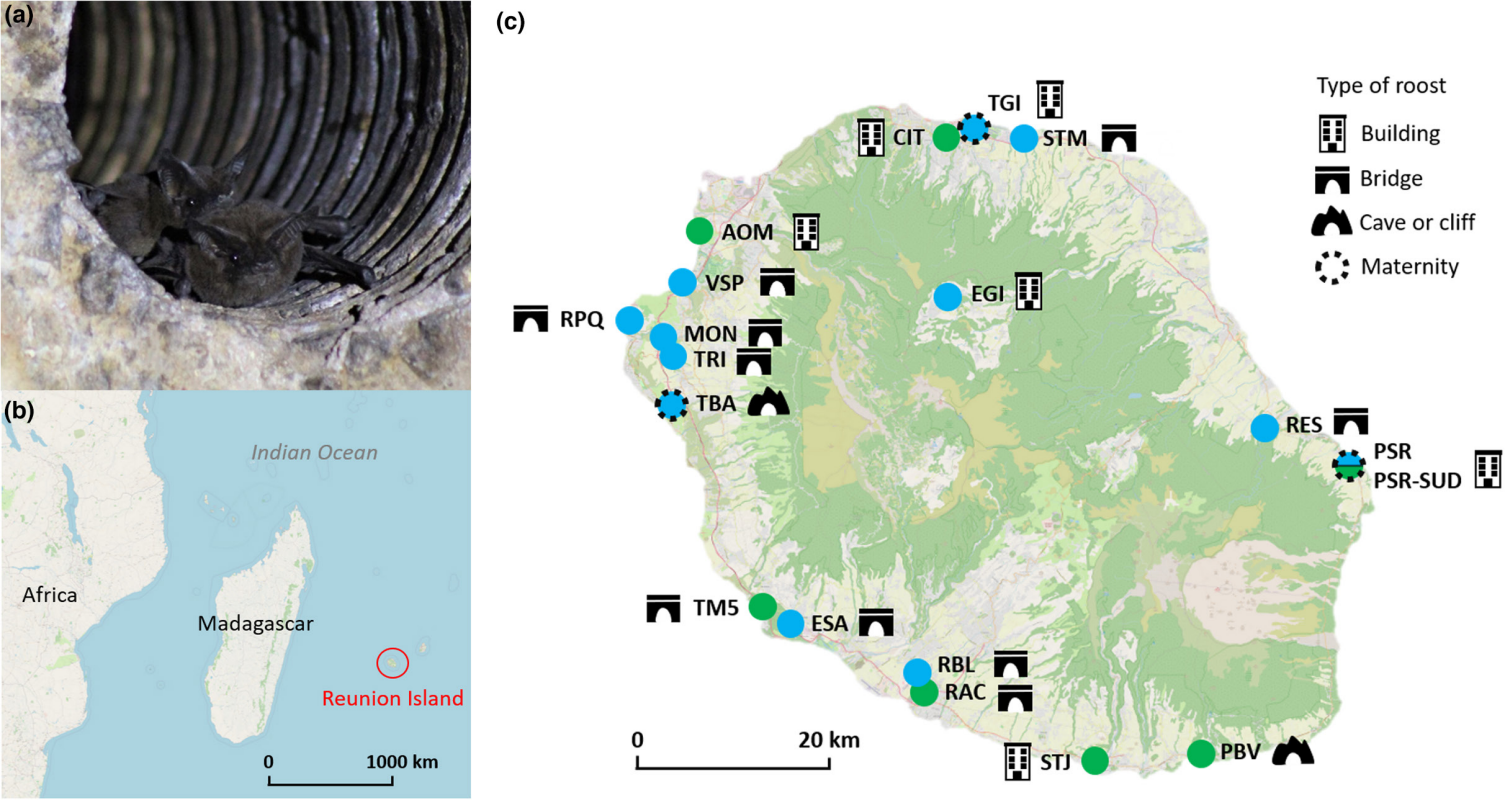
Sampling sites of the Reunion free‐tailed bat (*Mormopterus francoismoutoui*). (a) A photography of bats roosting inside a pipe in a bridge. (b, c) Location of Reunion Island and the studied roosts with the green color indicating forest areas and the pale indicating urbanized area. The twelve roosts in blue were monitored regularly over the 27‐month study while the seven roosts in green were sampled only once. PSR and PSR‐SUD roosts are located in the same building. Maps were created using Qgis (QGIS.org, 2022) with background of OpenStreetMap under License CC‐BY‐SA 2.0. Photo credit: Samantha Aguillon.

From October 2018 to December 2020 (27 months), we monitored 19 roosts of the Reunion free‐tailed bat, throughout the island (Figure 1). Roosts were investigated in various habitats including two roosts in natural settings (caves and cliffs) and 17 roosts in human constructions (7 roosts in buildings and 10 in bridges; Table 1). Roost name was coded with a 3‐letter code. Longitudinal monitoring was performed across successive “sampling periods” occurring every 3–8 weeks. To monitor all roosts within a study period, each sampling period lasted for 3–4 weeks (Table S1). Monitoring included both capture‐mark‐release of bats and roost size estimation.

**TABLE 1:** Roost characteristics of the Reunion free‐tailed bat (*Mormopterus francoismoutoui*).

Roost	Habitat	Roost size estimation		Number of sampling periods	Number of captured individuals					Mean male %
		Method	Minimum and maximum (number of estimations)		Total	Male	Female	Adult	Juvenile	
AOM	Building	Chamber	2560 (1)	1	57	33	24	35	22	57.89
CIT	Building	Joints	50 (1)	1	37	37	0	37	0	100
EGI	Building	At emergence	0–620 (2)	5	269	167	102	231	38	62.08
ESA	Bridge	Joints	570–3200 (15)	16	730 (+1 indetermined age)	349	381	651	78	47.81
MON	Bridge	Joints	197–1150 (16)	16	557	381	176	504	53	68.40
PBV	Cliff	At emergence	300 (1)	1	11	7	4	9	2	63.64
PSR	Building	–	–	16	615	480	135	586	29	78.05
PSR‐SUD*	Building	–	–	1	52	26	26	9	43	50
RAC	Bridge	Joints	153 (1)	1	16	12	4	16	0	75
RBL	Bridge	Joints	350–1300 (16)	16	734 (+1 indetermined age)	362	372	633	100	49.32
RES	Bridge	Chamber & joints	35–420 (9)	9	301	163	138	268	33	54.15
RPQ	Bridge	Joints	150–3000 (13)	16	601 (+1 indetermined age)	240	361	573	27	39.93
STJ	Building	At emergence	200 (1)	1	39	26	13	39	0	66.67
STM	Bridge	Chamber	27–350 (13)	13	451	336	115	428	23	74.50
TBA*	Cave	Covered surface	0–100,000 (12)	13	607	127	480	372	235	20.92
TGI*	Building	At emergence	14–1270 (35)	16	641	371	270	501	140	57.88
TM5	Bridge	Joints	200 (1)	1	30	17	13	0	30	56.67
TRI	Bridge	Joints	115–251 (5)	5	164	118	46	147	17	71.95
VSP	Bridge	Chamber & joints	15–6000 (17)	17	809	506	303	724	85	62.55
Total	–	–	–	165	6721	3758	2963	5763	955	–

*Note*: Maternity roosts (where parturition occurred) are indicated with an asterisk (*) next to the roost name. The number of sampling periods provide information on the monitoring frequency. The male percentage is calculated over all sampling periods.

### 2.2 Bat capture

In total, longitudinal captures were performed in 12 roosts (from 5 to 17 sampling periods depending on the roost, Table 1). Because of time and logistic constraints, the 7 remaining roosts were only sampled once, during known periods of roost occupation. For most sampling periods, the majority of bats (*n* = 4978, 74%) were captured during the dusk emergence, reaching a maximum of 60 individuals (over one or two consecutive nights if no or only few bats were captured during the first night). We mainly used harp traps (Faunatech Ausbat) and Japanese monofilament mist nets (Ecotone) installed close to the roost exit, without completely blocking the exit of bats. Because it was not possible to set up traps at the exit of some roosts, a butterfly net was also used by carefully approaching resting (nonflying) individuals during the day. After capture, bats were immediately hydrated using a sterile syringe and water, placed in a clean individual bag close to a warm source (hot water bottle), and processed at the capture site. To limit disturbance to bats at critical times of the year, trapping was not undertaken from the beginning of the parturition period (December) until the time there were no more pink‐colored newborns in the maternities (around mid‐January), nor on nonflying juveniles.

**FIGURE 2:**
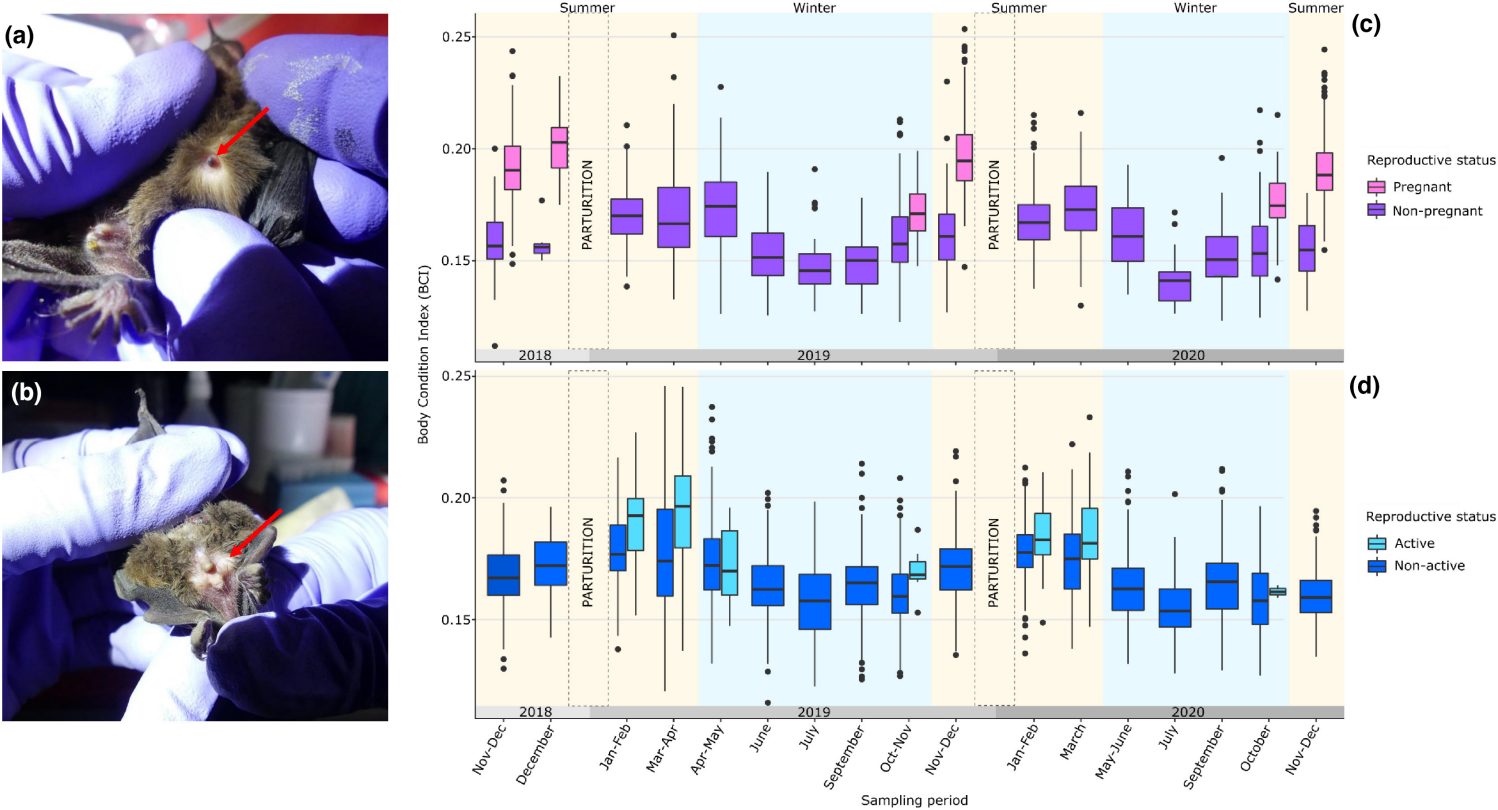
Visual indicators of the reproductive status of the Reunion free‐tailed bat and temporal variation of the Body Condition Index (BCI) according to the reproductive status. (a) Pregnant female with inflated nipples. (b) Male with large testes. (c) Temporal variation of adult female's BCI according to pregnancy status. (d) Temporal variation of adult male's BCI according to reproductive status (active or not). Summer and winter periods correspond to rainy and dry seasons and are indicated by yellow and blue backgrounds. Photo credit: Muriel Dietrich.

We visually determined the sex, age, and reproductive status of each individual. Age was determined by examining the epiphysis fusion in finger articulations that are not welded for juveniles. Juveniles were reasonably identifiable until July. After, it starts being difficult to see unfused finger joints, and some juveniles might have been classified as adults. Newborns were not captured but a visual inspection of the roosts, whenever possible, was performed to check for the presence of small size and pink‐colored individuals. Nonbreeding females were classified in accordance with the visibility of nipples: nonvisible (M0) or visible but noninflated (M1). Reproductively active females were recorded as pregnant, lactating, and postlactating. Pregnancy was determined by slight palpation of the abdomen, and the presence of inflated nipples (Figure 2a). Lactating females were defined when they had chewed inflated nipples (M2), and postlactating when hair regrowth was visible around the nipples, which were noninflated and chewed (M3). Males with large testes were considered reproductively active. It should be noted that testes are difficult to observe in this species (Figure 2b). For each bat, we also measured the forearm length with a caliper and the mass using an electronic scale for each bat. To identify all individuals, bats were tattooed using a dermograph with black ink on the right propatagium with an individual alphanumeric code. Finally, each individual bat was immediately released at the capture site after being processed.

Handling of bats was performed using personal protective equipment (FFP2 masks, nitrile gloves, as well as Tyvek suits and respirator cartridge masks inside the cave). Gloves were disinfected between each bat individual and changed regularly, and all the equipment was disinfected between sites as well. Bat capture and manipulation were evaluated by the ethic committee of Reunion Island, approved by the French Ministry of Research (APAFIS#10140‐2017030119531267), and conducted under a permit delivered by the Direction de l'Environnement, de l'Aménagement et du Logement (DEAL) of Reunion Island (DEAL/SEB/UBIO/2018–09).

### 2.3 Roost size estimation

A longitudinal monitoring (at least 5 different estimations) of the roost size was set up for 10 of the 12 monitored roosts (ESA, MON, RBL, RES, RPQ, STM, TBA, TGI, TRI, and VSP), because of the difficulty to count bats in PSR (no counts at all) and EGI (only 2 counts possible) buildings. For the nonmonitored roosts (AOM, CIT, PBV, RAC, STJ and TM5), roost size was estimated visually once over the study period, except for PSR‐SUD (no count at all). Roost size estimations were conducted either the day of a capture session or up to 14 days before or after capture, because of logistic constraints. To estimate roost size, different methods were used according to roost type (Table 1). Because this species emerges during the dusk when the sunset is not over, and light condition is favorable, bats were visually counted at emergence when possible. For the cave, because counting at emergence is challenging with the high number of bats, estimation was based on the size of the bat‐covered surface in m^2^, observed during the morning, multiplied by the visually assessed density of individuals (900 bats/m^2^), as in Héré et al. (2009). For bridges and some buildings with multiple exits, making counts at emergence was difficult and we rather visually counted during the day, when bats were resting bats and easily visible in joints and chambers.

### 2.4 Statistical analyses

Variation of roost size over time was investigated using a generalized additive mixed model (GAMM) with a quasi‐distribution and identity link, including the sampling period as a fixed effect, and the roost and roost size estimation method (joints or chambers/at emergence/covered surface) as random effects. When capture was undertaken over 2 days for the same roost, and thus roost size was estimated twice, we used the mean size calculated over the 2 days.

Sex‐ratio (with 95% confidence interval) was first estimated globally by calculating the proportion of males captured over the study in the 19 roosts. Bias differences were tested using a χ^2^ test. Temporal variation of the sex‐ratio was then investigated using a linear mixed model (LMM), including sampling period as a fixed effect and roost as a random effect. We also examined the influence of roost size (expressed as its logarithm) on sex‐ratio patterns using a Pearson correlation test. Finally, the sex‐ratio was estimated for adults and juveniles separately, and differences from a 1:1 ratio were tested for each sampling period, using *χ* ^2^ tests.

We investigated how adults of each sex aggregate or segregate among roosts. We used the Sexual Segregation and Aggregation Statistic (SSAS), a test comparing observed aggregation/segregation patterns to a random association of males and females (Bonenfant et al., 2007), and tested whether SSAS was correlated to the sex‐ratio using a Pearson correlation test. The test of SSAS estimates the confidence limits (CL) around the observed values among sampling periods, specific to each species (Bonenfant et al., 2007). By comparing SSAS values with the CL, we can determine if individuals are segregated (SSAS values above CL), aggregated (SSAS values below CL) or randomly associated (SSAS values in CL). To analyze if segregation patterns were positively correlated to the roost size, we calculated, for each sampling period, the global roost size (sum over all roosts, expressed as its logarithm), and the global SSAS and used a Pearson correlation test.

Reproductive phenology was first analyzed by identifying active periods in females (pregnancy, lactation, and postlactation) and males (large testes as a proxy of mating), as well as the presence of newborns and flying juveniles. Then, patterns of aggregation and segregation among roosts between adults and juveniles were examined using the SSAS. Age segregation was analyzed in correlation with the percentage of juveniles using a Pearson correlation test.

Sexual dimorphism occurrence was investigated first in adults, by testing the difference in forearm length between sexes, using a LMM including the roost as a random effect to account for potential geographic variation (excluding data for recaptured bats to avoid redundant data). Sexual dimorphism in juveniles was also analyzed by measuring the growth of the forearm over time using a LMM including sex and date as fixed effects (and the interaction), and roost and year as random effects (excluding recaptured bats) to control for potential difference of juveniles growth between years. The relationship between the forearm length and the mass, including adults and juveniles, was tested using a Pearson correlation test.

Subsequently, the individual body condition was estimated using the Body Condition Index (BCI) by calculating the mass/forearm length ratio. Variations in BCI were investigated using a LMM including age and sex (and the interaction) as fixed effects and roost as random effects, followed by Tukey's post hoc tests. Sampling period was also added as a random effect to accommodate for potential within‐year variations. Then, BCI was analyzed in adults using a LMM in relation to the reproductive status (active or not; as a fixed effect), also including roost and sampling period as random effects. Analyses were performed in adult females and adult males, separately. Because aggregation phenomenon (increase in roost size) can represent a cost or a benefit for the fitness of individuals (Brown, 2016), we tested if the roost size could affect the body condition of reproductive individuals. We focused the analysis on the pregnancy period in females (November–December) and on the mating period for males (March, see results). BCI variation was analyzed using a LMM in relation to the roost size (expressed as its logarithm) and reproductive status (and the interaction) as fixed effects, including the roost as a random effect. We also included the year and date as nested random effects to accommodate for temporal variations of the BCI within the pregnancy and mating periods.

All analyses were performed in Rstudio v.1.4.1106 (Rstudio Team, 2020) using packages *date*, *dplyr*, *emmeans*, *ggplot2*, *lme4*, and *mgcv*. For LMM analysis, the most parsimonious model was selected by excluding step by step every nonsignificant variable of the complete model and using an ANOVA test to compare models. All the models used are presented in Table S2.

## 3 RESULTS

### 3.1 Temporal evolution of roost size

**FIGURE 3:**
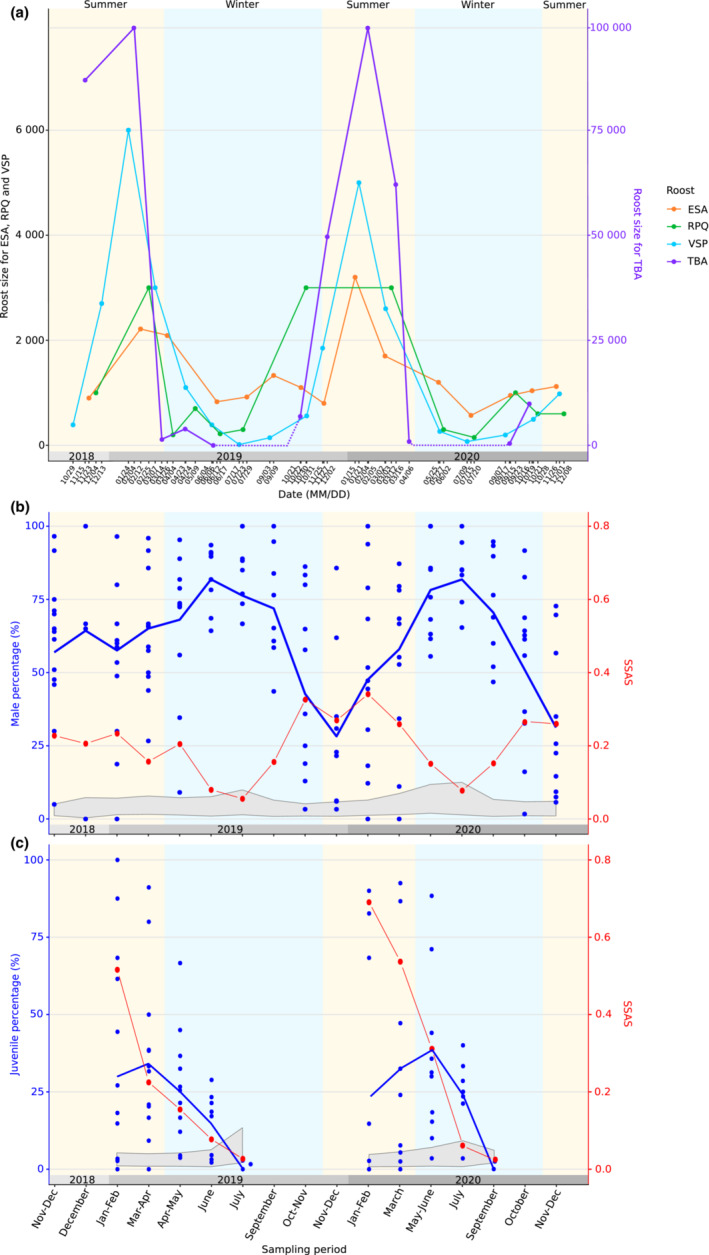
Temporal variation of (a) roost size and (b, c) segregation patterns of the Reunion free‐tailed bat. (a) For better visualization, roost size is only presented for the four largest roosts. Data for the other roosts can be found in Figure S1. Roost size scale is presented on both axes because of large differences for the TBA roost size. The scale for TBA roost is indicated on the right of the graph (in purple) and portion of dotted line correspond to the period when the cave is known to be empty based on earlier studies (Dietrich et al., 2015). (b, c) Sexual Segregation and Aggregation Statistic (SSAS) was calculated per sampling period, when the value is up to the gray zone it indicates that bats are segregated and when value is within the gray zone it indicates that bats are randomly associated in roosts. In (b) SSAS represents the segregation between sexes in adults. For each sampling period, male percentage in the multiple roosts (blue points), and its mean (continuous blue line) are also indicated. In (c) SSAS represents the segregation between age classes (adults vs. juveniles). Juveniles cannot be reasonably distinguished after June. For each sampling period, the percentage of juveniles is also represented for the multiple roosts and the continuous line represents the mean. Summer and winter periods correspond to rainy and dry seasons and are indicated by yellow and blue backgrounds.

Over a 27‐month period, we gathered 200 estimates of roost size for 17 different roosts. Estimates of roost sizes were from 0 to 100,000 individuals (details in Table 1). A marked temporal variation of the roost size was found (GAMM_1_: *p* < .001; Table S2). In particular, a synchronized increase in roost size during austral summer, starting in October and reaching a maximum around January and February, was observed (Figure 3a for biggest roosts and Figure S1 for the others). Increase in roost size during summer was particularly massive in the TBA and VSP roosts; in TBA, the number of bats went from 0 to 100,000, and in VSP the roost size increased by a factor of 400 (Figure 3a). During austral winter, low numbers of bats (sometimes none) were generally observed, except in TGI and MON roosts where larger numbers were present (Figure S1).

### 3.2 Sex‐ratio and sexual segregation

**FIGURE 4:**
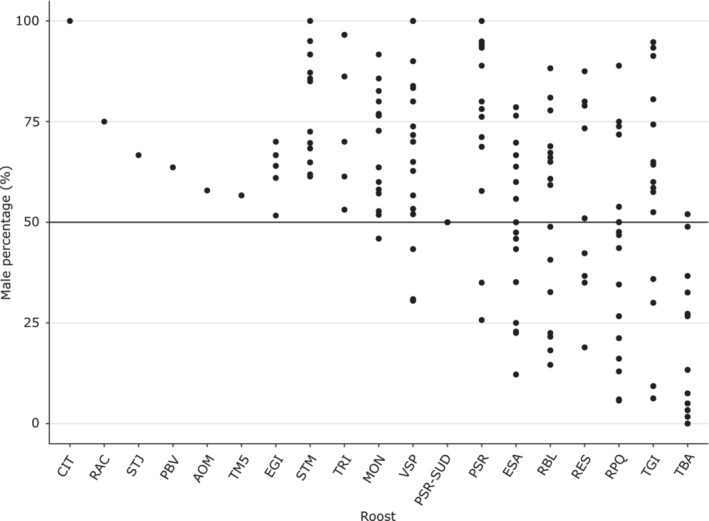
Sex‐ratio variation between roosts. For each roost, multiple points represent the percentage of males at different sampling periods. For 7 roosts, they were sampled only once and only one point is represented.

**FIGURE 5:**
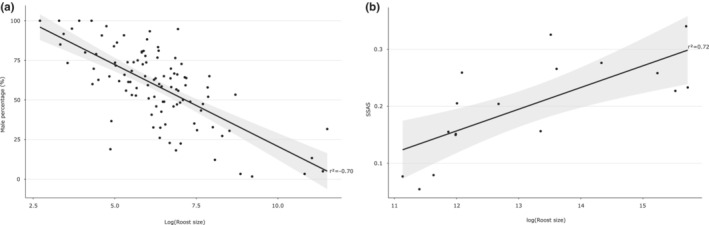
Correlation of the roost size (expressed as its logarithm) with (a) the sex‐ratio and (b) the Sexual Segregation and Aggregation Statistic (SSAS) in adults.

Over the 17 sampling periods, we captured 6721 bats in 19 roosts (Table 1). The global sex‐ratio was male‐biased (56 ± 1.2% of males; $\chi_{1}^{2}$ = 46.95, *p* < .001), and varied among roosts (LMM_2_: $\chi_{16}^{2}$ = 71.51, *p* < .001) as well as over sampling periods (LMM_2_: $\chi_{16}^{2}$ = 92.46, *p* < .001; Figure 4 and Table S2). However, this male‐biased sex‐ratio was driven by adults (57 ± 1.3% of males; $\chi_{1}^{2}$ = 58.49, *p* < .05) since sex‐ratio was at equilibrium in juveniles (49 ± 3.2% of males; $\chi_{1}^{2}$ = 0.28, *p* > .05). Most of the studied roosts harbored a majority of males all year long, although others have hosted either more males or more females, and even a balanced sex‐ratio, depending on the time of the year (Figure 4). A female‐biased sex‐ratio was observed in a single roost (TBA), being the largest maternity roost (where parturition occurred) known on the island. Similarly, we found that larger roost size was significantly associated with a lower percentage of males within roosts (*r* ^2^ = −0.70, *p* < .001; Figure 5a).

We found that the sex‐ratio was correlated to the segregation pattern between sexes as measured by the SSAS (*r* ^2^ = −0.84, *p* < .001; Figure S2). Indeed, the analysis of sex‐ratio in adults over time revealed a significant female‐biased sex‐ratio in summer (from October to December; Figure 3b and Figure S3), which also correlated to a clear segregation pattern between sexes at this period, highlighted by the increase in the SSAS. In contrast, in winter (June and July), the percentage of males was particularly high, and males and females were more randomly aggregated within roosts. Consequently, the segregation pattern observed between sexes was also positively associated with larger roost sizes (*r* ^2^ = 0.72, *p* < .001; Figure 5b).

### 3.3 Reproductive phenology

**FIGURE 6:**
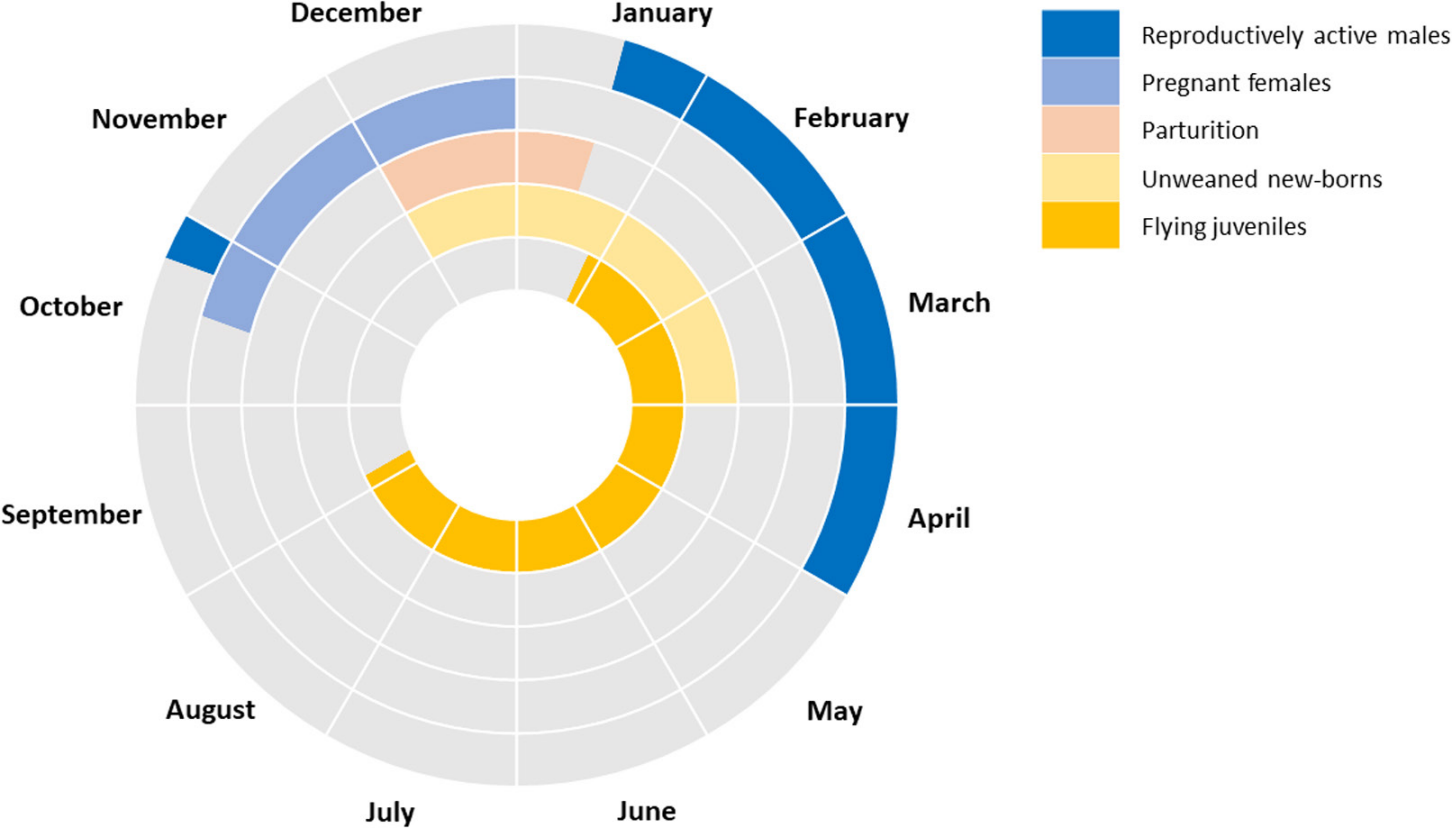
Hypothesized reproductive cycle of *Mormopterus francoismoutoui* based on a fine‐scale monitoring of 19 roosts in Reunion Island over a 27‐month period.

A total of 201 reproductively active males (with large testes) were captured. Most (95%) were synchronously observed from late January to April, both in 2019 and 2020 in 12 roosts, suggesting that the mating period probably occurred during these months (Figure 6). A few reproductively active males (5%) were also observed in October both years. A high percentage of reproductively active males were observed in male‐biased sex‐ratio roosts (MON, PSR and STM).

Pregnant females represented 14% of total captured bats and were observed from October to December, in almost all roosts sampled at this period (*n* = 11). The three roosts with the highest percentage of pregnant females in the November–December sampling period were TBA, TGI, and RPQ, reaching 86% of captured individuals in TBA and TGI (in 2018 and 2020 respectively), and 92% in RPQ (in 2019). Despite the fact that pregnant females were spread across roosts, parturition occurred in just a few roosts: TBA, TGI, and PSR‐SUD, based on the observation of newborns from early December to late January. After the parturition break for captures (December to mid‐January), we caught lactating and postlactating females in 11 roosts until March.

Although parturition occurred only in a few roosts, flying juveniles were observed in almost all roosts from January to September (Figure 6, Figure S4 and Table 1), with the highest numbers in TBA and TGI roosts (39% and 22% of juveniles captured over the study, respectively). Flying juveniles were captured 39 and 46 days after the first observation of parturition, in 2019, and 2020, respectively. The SSAS showed that juveniles and adults were particularly segregated in January and February, and then tended to be randomly distributed toward winter months (Figure 3c). However, this segregation pattern between ages was not correlated to the percentage of juveniles in the bat population (*r* ^2^ = 0.58, *p* > .05, Figure 3c). Finally, we can notice that TM5 (sampled mid‐February) was a temporary roost, only composed of presumably recently weaned juveniles (Figure S4).

### 3.4 Sexual dimorphism and body condition

Although the difference was subtle, adult males had a significant longer forearm (mean = 39.28 mm, SE = 0.013), as compared to adult females (mean = 39.19 mm, SE = 0.015) (LMM_3_: $\chi_{1}^{2}$ = 21.40, *p* < .001; Figure S5 and Table S1). The analysis on juveniles suggested the same pattern (LMM_4_: $\chi_{1}^{2}$ = 4.84, *p* = .03) and also showed forearm length increasing with time (LMM_4_: $\chi_{1}^{2}$ = 18.13, *p* < .001, Figure S6). Furthermore, the relation between the forearm length and the mass was significantly positive (*r* ^2^ = 0.21, *p* < .001; Figure S7).

The BCI value of individuals was strongly dependent on age and sex, with an interaction between both variables (LMM_5_: $\chi_{1}^{2}$ = 22.73, *p* < .001; Table S2). As expected, adults had a higher BCI value than juveniles (LMM_5_: $\chi_{1}^{2}$ = 906.93, *p* < .001). In adults, females showed a higher BCI value than males (Tukey's post hoc test: *p* = .02), while in juveniles, males were in better condition (Tukey's post hoc test: *p* = .02, Figure S8). Analysis over time revealed a strong variation of BCI for adults (Figure 2c,d): females showed an increase BCI during November–December months corresponding with pregnancy (LMM_6_: $\chi_{1}^{2}$ = 605.36, *p* < .001), whereas in males, the increase occurred later around March (LMM_7_: $\chi_{1}^{2}$ = 445.27, *p* < .001; Table S2). This increase was associated with a significant positive effect of the reproductive status for both females (LMM_6_: $\chi_{1}^{2}$ = 349.13, *p* < .001) and males (LMM_7_: $\chi_{1}^{2}$ = 103.61, *p* < .001; Table S2). For both sexes, a continuous decrease of the BCI was then observed during winter (Figure 2c,d).

**FIGURE 7:**
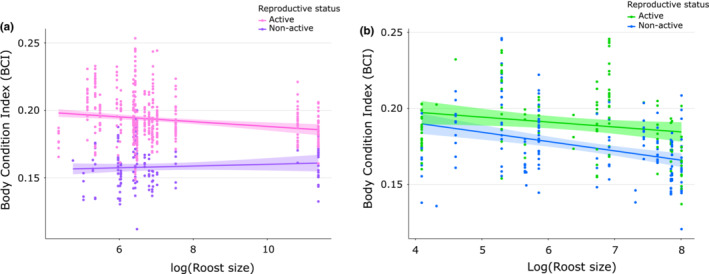
Correlation between the body condition (BCI) and the roost size (expressed as its logarithm) in (a) adult females according to their reproductive status during the pregnancy period (active status includes pregnant and a few lactating females), and (b) adult males according to their reproductive status during mating period (active status corresponds to males with large testes).

Analysis throughout the pregnancy period showed a negative effect of roost size on the BCI, only for reproductively active females (LMM_8_: $\chi_{1}^{2}$ = 5.23, *p* = .02; Table S2), which were thus in better condition in smaller roosts (Figure 7a). However, this result was no longer significant when the same analysis was performed excluding the large maternity roost of TBA (LMM_9_: $\chi_{1}^{2}$ = 0.001, *p* = .97; Table S2). For males, analysis throughout the mating period showed no significant correlation between the roost size and the BCI of active or nonactive males (LMM_10_: $\chi_{1}^{2}$ = 1.09, *p* = .29; Figure 7b and Table S2).

## 4 DISCUSSION

Based on an intensive and fine‐scale study of multiple roosts, we revealed a highly dynamic roosting behavior and phenology of the Reunion free‐tailed bat. Longitudinal monitoring first confirmed the large population size of this bat species, probably far exceeding 120,000 individuals all over the island, and representing a mean density of bats of at least 48 bats/km^2^. This relative large population size could result from the low number of indigenous predators, which is typically encountered in small oceanic islands (Lomolino et al., 2013). The Reunion free‐tailed bat could also take advantage of the growing urbanization of the island, which creates attractive areas with a huge variety of suitable roosts in terms of volume and configuration (such as chambers within bridges) (Goodman et al., 2008). In addition, this bat species is likely experiencing low competition for resources, as the only another insectivorous bat present on the island, the Mauritian tomb bat, is far less abundant (O'Brien, 2011). Despite such a large population size, the Reunion free‐tailed bat is facing displacement from its roosts, as its excrement and musky smell leads to considerable nuisance for humans (Augros et al., 2015). Significant effort has been devoted to roost translocation strategies, but because of mitigated results due to unsuccessful operations, such roost disturbance may represent a threat for the conservation of this bat species.

Over the 19 studied roosts, a strong heterogeneity in roost size and sex‐ratio was observed, suggesting a complex social organization and distinct functions of the different roosts. The population is composed of a continuum of roosts, encompassing male, mixed, maternity (female‐biased) roosts, and juvenile roosts, all probably interconnected by important seasonal movements. Although we cannot exclude a higher mortality in females, we rather think that the global male biased sex‐ratio found in this study is linked to a sampling bias toward adult males because adult females seem spatially and temporally restricted to a few roosts (see below) and were difficult to capture during winter especially. The even sex‐ratio observed in juveniles adds support to this hypothesis.

The strong heterogeneity in roost size and sex‐ratio was characterized by marked seasonal variation. In the majority of roosts, cyclic and synchronized occupation peaks were recorded during austral summer, coinciding with a global female‐biased sex‐ratio during this period. This is coherent with a widespread aggregation of pregnant females within roosts, and segregation between males and females. Such aggregation behavior is commonly observed in bats, for example, in the Brazilian free‐tailed molossid *Tadarida brasiliensis*, which forms the largest maternity roosts in the mammal world (McCracken & Wilkinson, 2000). Seasonal sexual segregation has also been documented in many bat species, especially temperate bats (Encarnação, 2012; Katsis et al., 2021) but also tropical bats (Cheng & Lee, 2004; Llaven‐Macías et al., 2021) such as the Madagascar sucker‐footed bat *Myzopoda aurita* (Ralisata et al., 2010). Such roosting behavior has been associated with different thermoregulatory strategies and energetic requirements, or even competition for food (Encarnação, 2012; Katsis et al., 2021; Levin et al., 2013). Here, we found that the body condition of pregnant females decreased in larger roosts (although this relationship was driven by the big TBA maternity roost). Our results thus suggest some costs of aggregation for pregnant females, with for example a higher parasitism and infection risk (Lourenço & Palmeirim, 2007; Lučan, 2006; Reckardt & Kerth, 2009). Indeed, previous studies in the Reunion free‐tailed bat showed an increased prevalence in bacterial and viral infections during the pregnancy period (Dietrich et al., 2015; Joffrin et al., 2022).

Surprisingly, while pregnant females were observed in almost all roosts, we only identified three maternities where parturition occurred synchronously (TBA, TGI, and PSR‐SUD). These results suggest dispersal of late‐pregnant females for parturition, as seen in other bat species, such as the Soprano pipistrelle *Pipistrellus pygmaeus*, which moves to the maternity roosts a few days before parturition (Bartonička & Gaisler, 2007). In several bat species, energy conservation is an important selective pressure for pregnant females which select warmer roosts, thus reducing costs of maintaining normothermy or forced torpor (López‐Baucells et al., 2016; Lourenço & Palmeirim, 2004; Sedgeley, 2001; Vonhof & Barclay, 1996). Interestingly, the three maternity roosts in our study were located in a huge natural cave where bat density is very high, in an electric building (releasing heat), and under a sheet metal roof (accumulating heat during the day), all of these conditions probably offering warm conditions. Deciphering the environmental conditions of these maternity roosts would help understanding microhabitat preferences in the Reunion free‐tailed bat, critical for effective conservation.

We observed a segregation pattern between adults and juveniles, particularly high in January and February. This may be explained by the fact that parturition occurred only in a few roosts, and that lactating females likely moved to other day‐roosts than the maternity roosts, at least when their youngs are already a few weeks old. Indeed, lactating females we caught, from mid‐January until March, were observed in 11 roosts, and for 9 of them, no parturition occurred. It is important to notice that around mid‐January, youngs were probably already a few weeks old. This suggests that, after a few weeks after birth, lactating females switched day‐roosts and went back to maternity roosts at night to suckle their nonflying youngs until weaning. Roost switching behavior can result from an adaptation of female bats to avoid parasite infestation (Bartonička & Gaisler, 2007). Indeed, although not measured in this study, high levels of ectoparasites on bats were observed at the time of parturition, particularly on newborns. Our data also suggest that juveniles of the Reunion free‐tailed bat started dispersing rapidly from maternity roosts—about 6 weeks after birth, which is consistent with the decrease over time of the segregation pattern between adults and juveniles. In the molossid bat *T*. *brasiliensis*, juveniles begin to fly after 5–6 weeks and are weaned 2 weeks after the first flight (Boero et al., 2020). Radio‐tracking of individuals, associated with the analysis of parasitism/infection levels, are necessary to better understand roost switching and dispersal behavior of the Reunion free‐tailed bat.

The reproductive cycle of males of the Reunion free‐tailed bat has never been documented. Surprisingly, our study revealed that males have large testes synchronously in all roosts around March. Theses males were in better condition than nonreproductively active males, which could be the consequence of an accumulation of energy reserve for mating to support the reproduction cost (Racey & Entwistle, 2000). Together, these results suggest that mating behavior likely occurred around March in the Reunion free‐tailed bat, a considerable time from the parturition period, and thus implying a long interruption of the reproductive cycle (about 7 months between mating and the start of pregnancy). This phenomenon is commonly observed in temperate bats, where mating occurs in autumn before hibernation, while parturition is in summer, as shown in the greater mouse‐eared bat *Myotis myotis* and Natterer's bat *Myotis nattereri* (Pfeiffer & Mayer, 2013). Several mechanisms can be involved in reproductive delay like sperm storage, delayed implantation or delayed development of the embryo (Crichton, 2000; Pfeiffer & Mayer, 2013), and can be linked to daily torpor in tropical bat species (Racey & Entwistle, 2000). The reproductive cycle of the Reunion free‐tailed bat seems to be similar to the south‐eastern free‐tailed bat *Mormopterus planiceps*, a molossid living in southeast Australia, having a single mating per year with sperm storage (Racey & Entwistle, 2000).

However, some males with large testes were also observed in October (coinciding with the beginning of the pregnancy period), and thus suggesting a second mating event, potentially in response to receptive females failing to fertilize in March. Such behavior has been documented for the European brown long‐eared bat *Plecotus auritus* males that could fertilize females after hibernation by storing sperm until spring (Pfeiffer & Mayer, 2013). In temperate regions, autumn swarming in specific underground sites is thought to be linked to mating (Furmankiewicz & Altringham, 2007; Kerth et al., 2003). Here, we observed males with larger testes in all the studied roosts, but this does not mean that mating is widespread among all roosts. The use of infra‐red cameras to study bat behavior within roosts may help identifying mating sites, which has crucial implications for conservation (Furmankiewicz, 2016; Neubaum & Siemers, 2021; Piksa & Bogdanowicz, 2011).

The end of austral summer was characterized by a decrease in roost size and a male biased sex‐ratio, suggesting that individuals, mainly females, leave the studied roosts. In addition, during austral winter, the body condition for both males and females drastically decreased, which may be linked to some limitation in food resources and the use of daily torpor. This mechanism reduces metabolism and body temperature in response to a reduced food availability during winter (Racey & Entwistle, 2000). Even if occupancy in some roosts remains important during winter, we hypothesize that bats, and especially females, live in small groups or even solitary during winter (Augros et al., 2015), especially because we have never observed large groups of females during winter. Reunion island is a small oceanic island, and thus straight distances possibly traveled by bats are probably less than 75 km (longest diagonal). However, the island is highly mountainous (highest peak standing at 3070 m), offering possibilities for short‐distance movements. For example, one natural roost was observed at 2070 m in October (Sanchez & Probst, 2013), showing that this bat species is able to live in high‐elevation roosts. Although altitudinal bat migration appears more common in bats in the temperate regions than in the tropics, few cases have been described in tropical species (McGuire & Boyle, 2013). For example, in the lesser long‐nosed bat *Leptonycteris yerbabuenae*, after juveniles start flying, females move to higher elevations to search for more resources. These migrations are sex biased as males remain in the southern portion of the range until mid‐summer, and join the reproductive females at the northern high‐elevation sites only in the autumn (Cockrum, 1991). Whether the Reunion free‐tailed bat uses the same migration strategy is unknown, but further telemetry and genetic studies of bats sampled at higher altitudes will help test this hypothesis and eventually identify wintering roosts (Johnson et al., 2015, 2017; Michaelsen et al., 2013; Moussy et al., 2013).

Our study is the first comprehensive description of the biology of the Reunion‐free tailed bat based on extensive capture data. The fine‐scale sampling scheme implemented here was necessary to reveal the complex social organization in this bat species. The Reunion free‐tailed bat provides a relevant example of how dynamic are sex‐specific behavioral strategies in bats, even for tropical species living in small oceanic islands. Information provided by our study will be critical to the delineation of effective conservation plans, especially in the context of growing urbanization and agriculture expansion in Reunion Island. Further investigations including bat tracking, roosting conditions, and genetic studies are warranted to better understand the seasonal and spatial movements of individuals within the island. Our study highlights that fine‐scale monitoring of island bat populations is crucial to help conservation and management of these highly threatened mammal species.

## AUTHOR CONTRIBUTIONS

**Samantha Aguillon:** Conceptualization (equal); formal analysis (lead); investigation (equal); visualization (lead); writing – original draft (lead); writing – review and editing (equal). **Gildas Le Minter:** Investigation (equal); writing – review and editing (equal). **Camille Lebarbenchon:** Funding acquisition (equal); investigation (equal); writing – review and editing (equal). **Axel O. G. Hoarau:** Investigation (equal); writing – review and editing (equal). **Céline Toty:** Investigation (equal); writing – review and editing (equal). **Léa Joffrin:** Investigation (equal); writing – review and editing (equal). **Riana V. Ramanantsalama:** Investigation (equal); writing – review and editing (equal). **Stéphane Augros:** Investigation (equal); writing – review and editing (equal). **Pablo Tortosa:** Funding acquisition (equal); investigation (equal); writing – review and editing (equal). **Patrick Mavingui:** Supervision (equal); writing – review and editing (equal). **Muriel Dietrich:** Conceptualization (lead); formal analysis (equal); funding acquisition (equal); investigation (lead); supervision (equal); validation (lead); writing – review and editing (equal).

## CONFLICT OF INTEREST STATEMENT

We have no competing interests to declare.

## Supporting information


Figure S1

Figure S2

Figure S3

Figure S4

Figure S5

Figure S6

Figure S7

Figure S8

Table S1

Table S2

## Data Availability

Data and analysis script are archived on Zenodo (with embargo until February 1st 2024) (https://doi.org/10.5281/zenodo.7540372).
