# Supplementary figures and images for: A population in perpetual motion: Highly dynamic roosting behavior of a tropical island endemic bat
Source: Ecol Evol. 2023 Feb 11;13(2):e9814. doi: 10.1002/ece3.9814 (PMC9919472; doi:10.1002/ece3.9814)

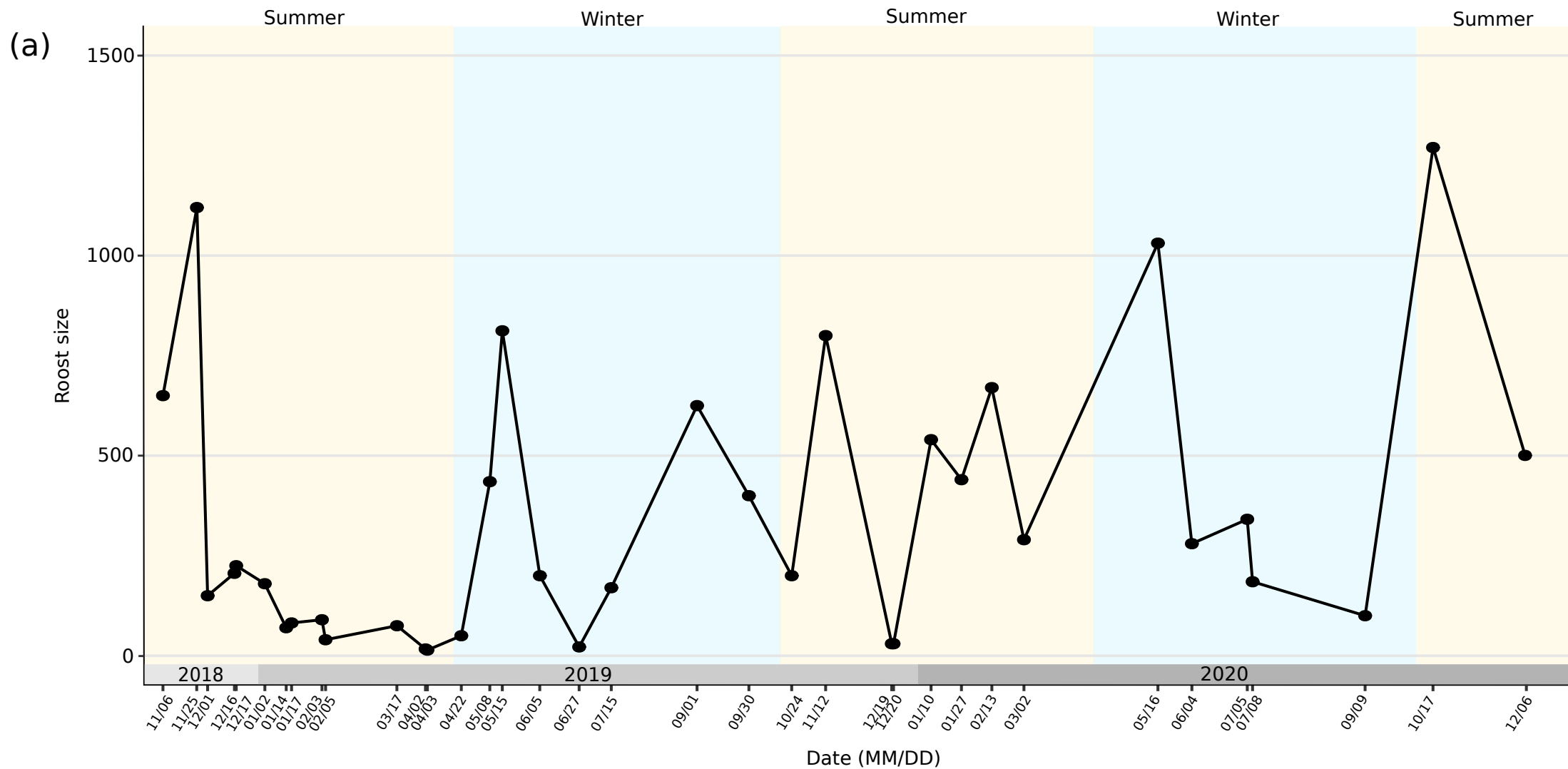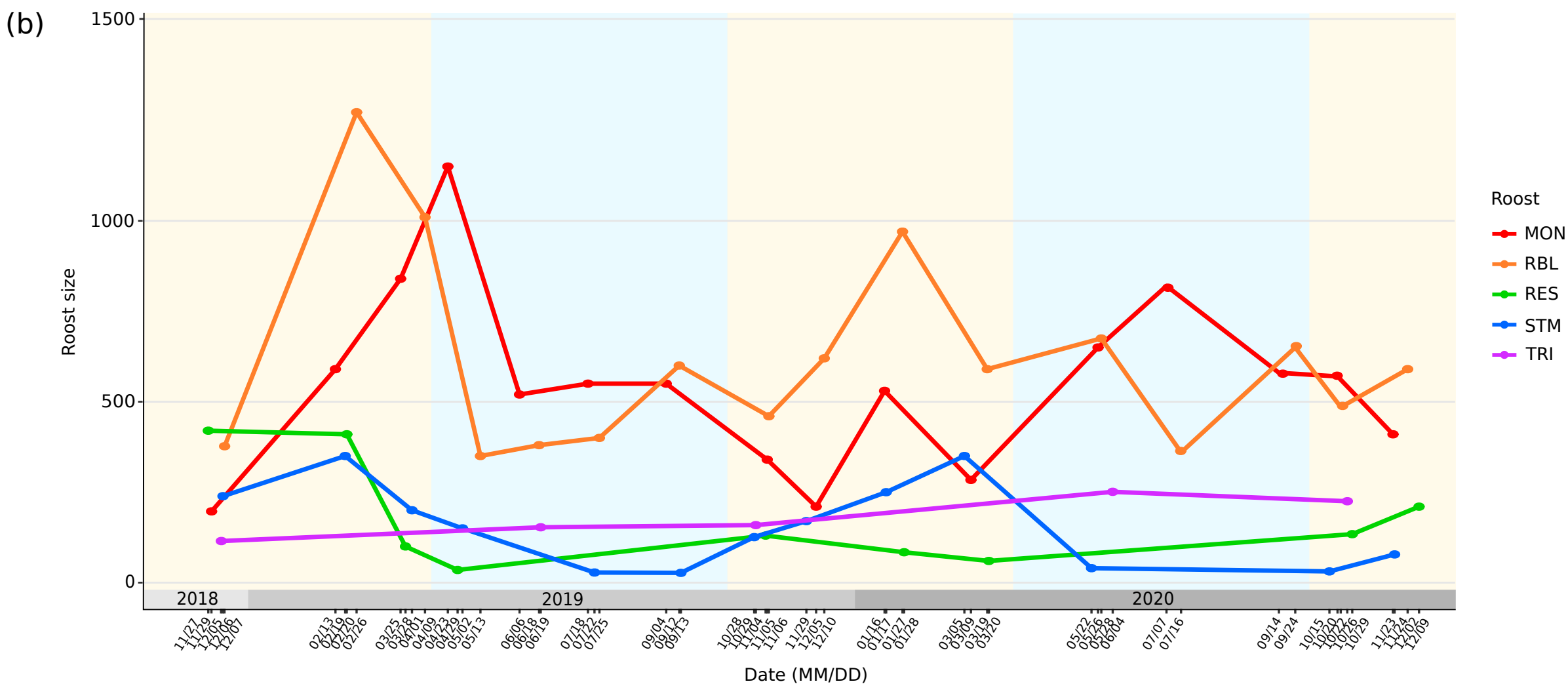

Supplement: Supplementary file 1 — Figure S1 [file ECE3-13-e9814-s007.pdf]

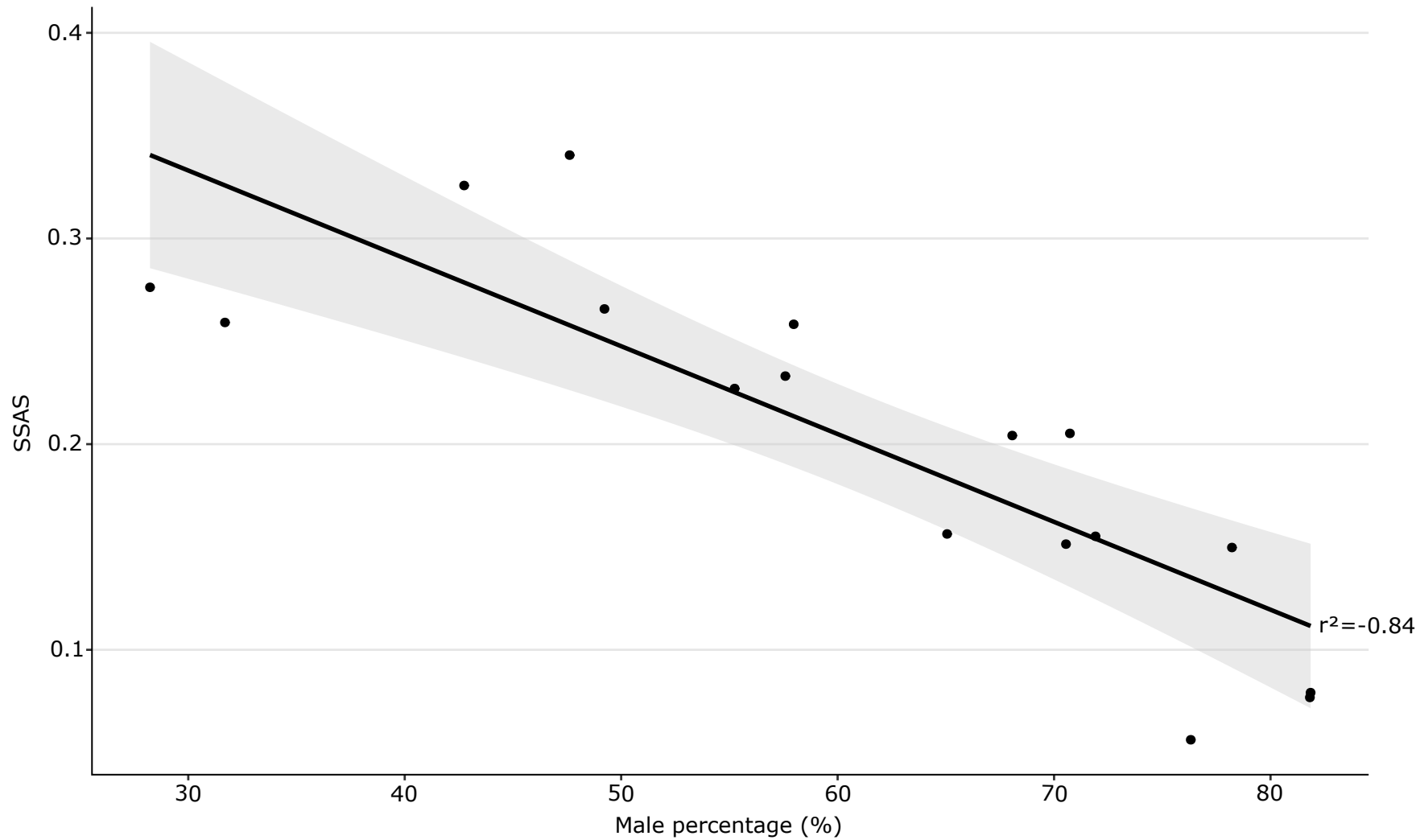

Supplement: Supplementary file 2 — Figure S2 [file ECE3-13-e9814-s004.pdf]

Number of captured bats

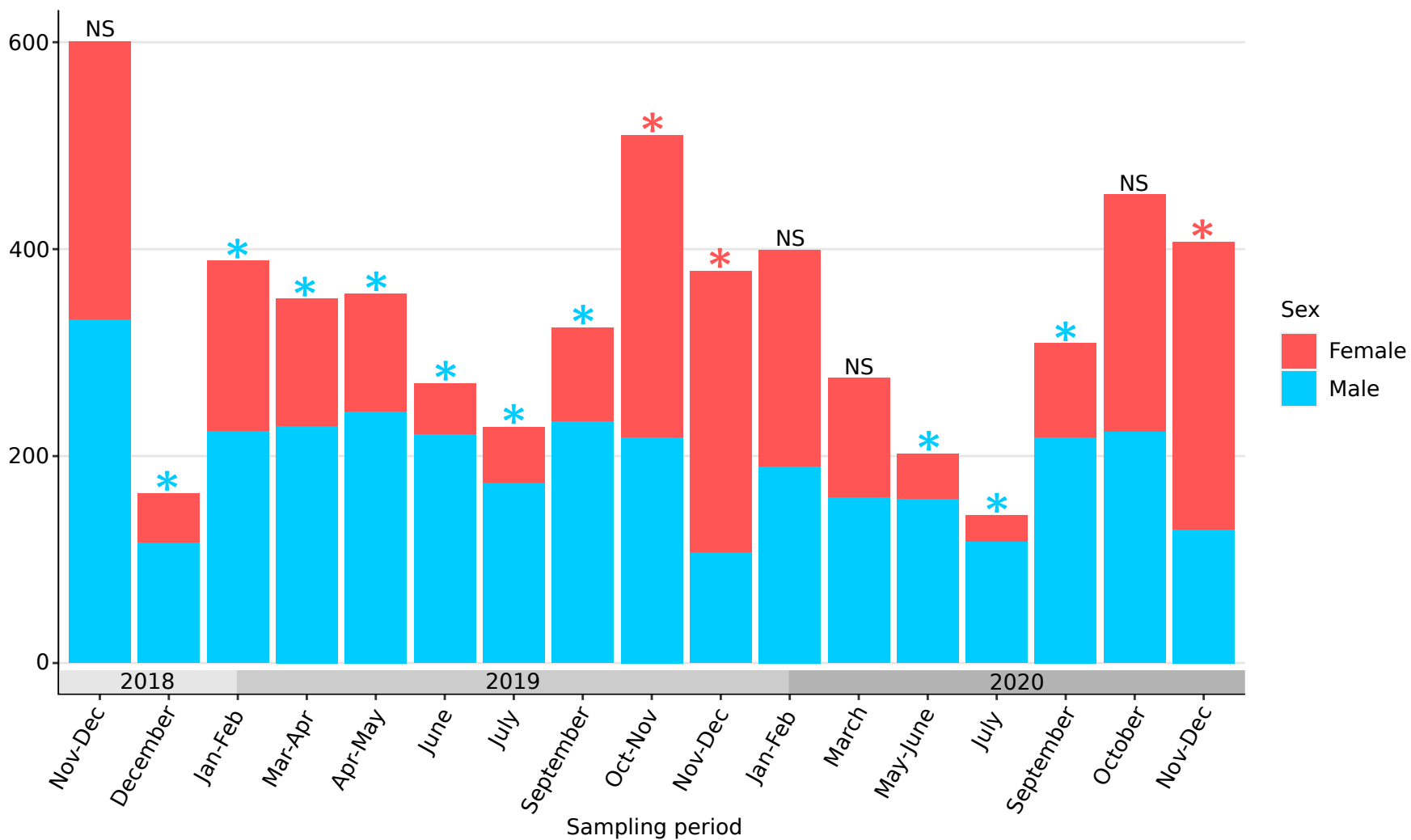

Supplement: Supplementary file 3 — Figure S3 [file ECE3-13-e9814-s002.pdf]

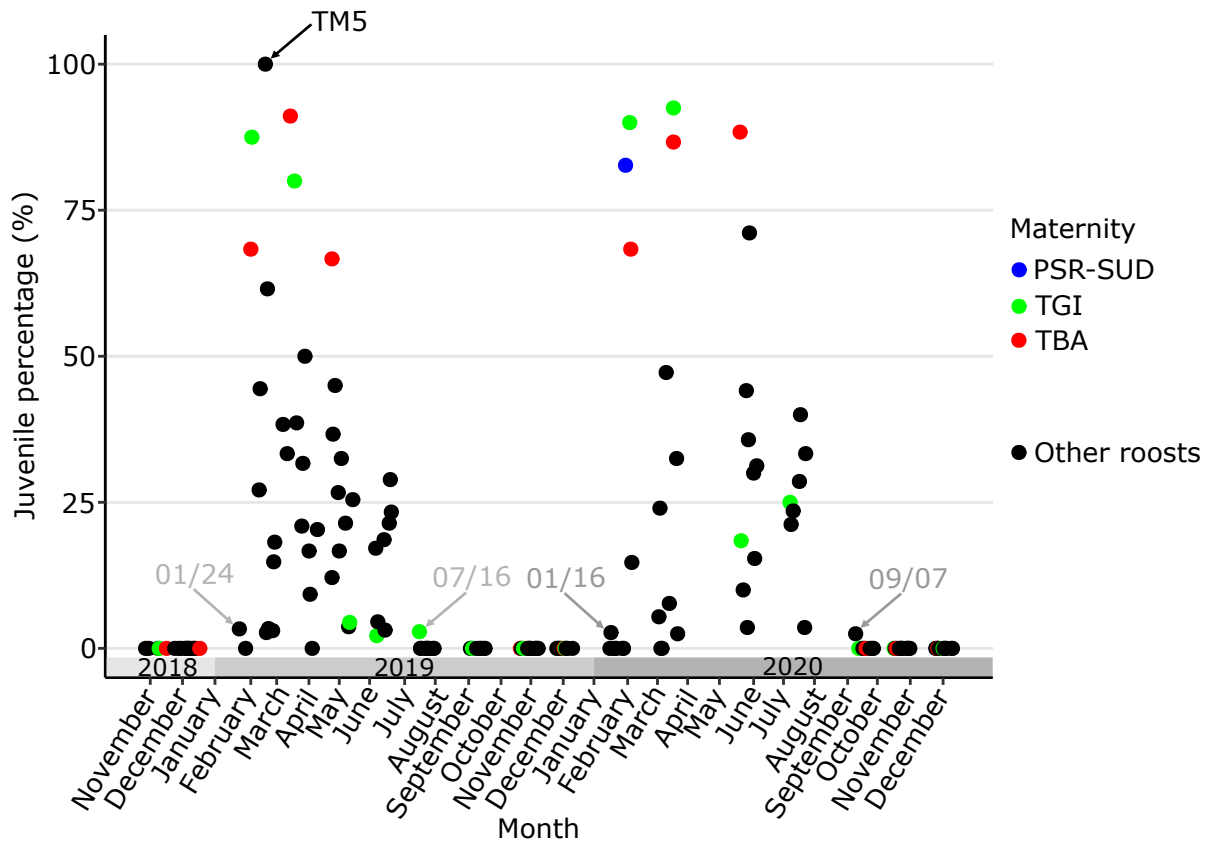

Supplement: Supplementary file 4 — Figure S4 [file ECE3-13-e9814-s005.pdf]

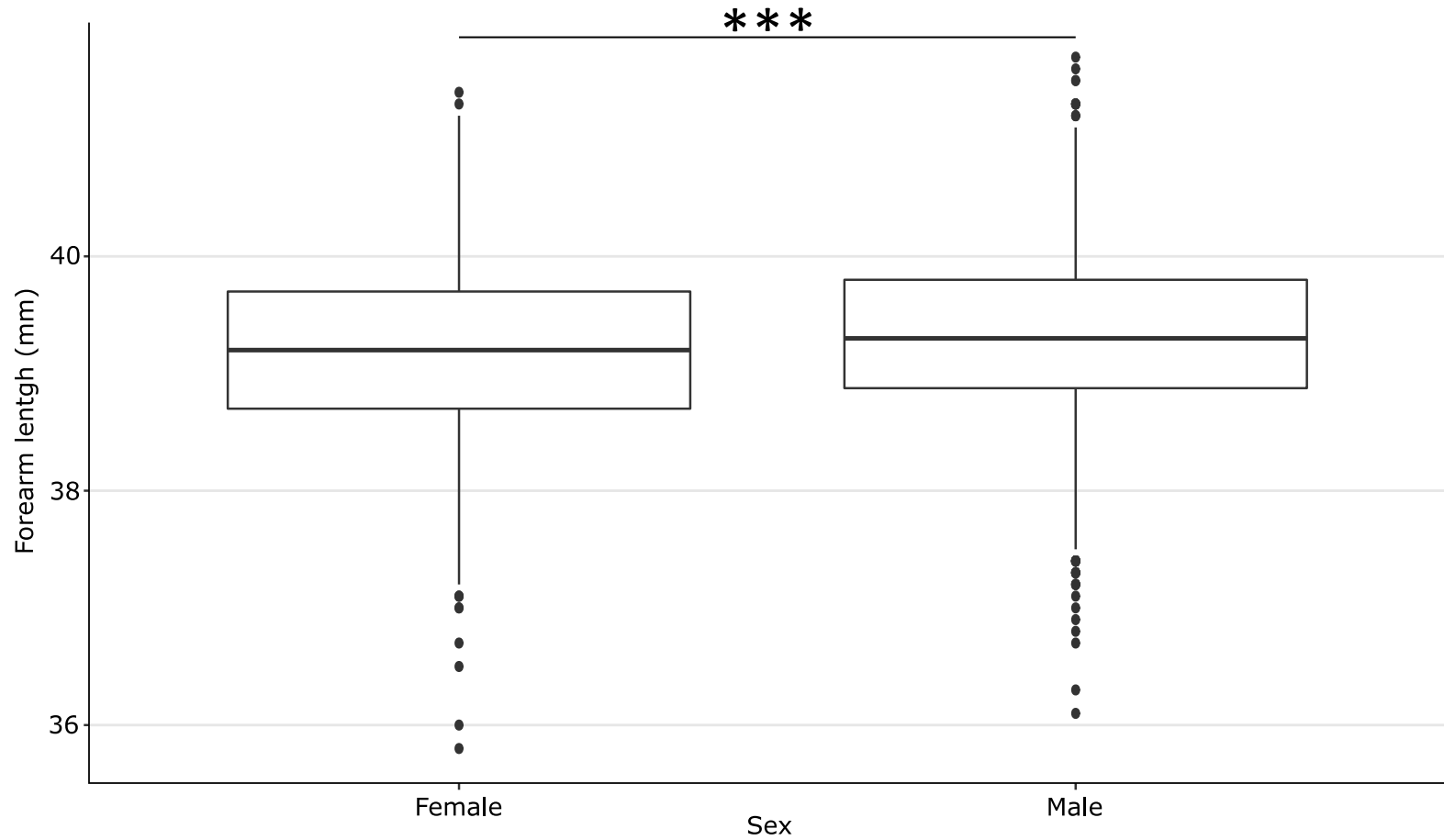

Supplement: Supplementary file 5 — Figure S5 [file ECE3-13-e9814-s001.pdf]

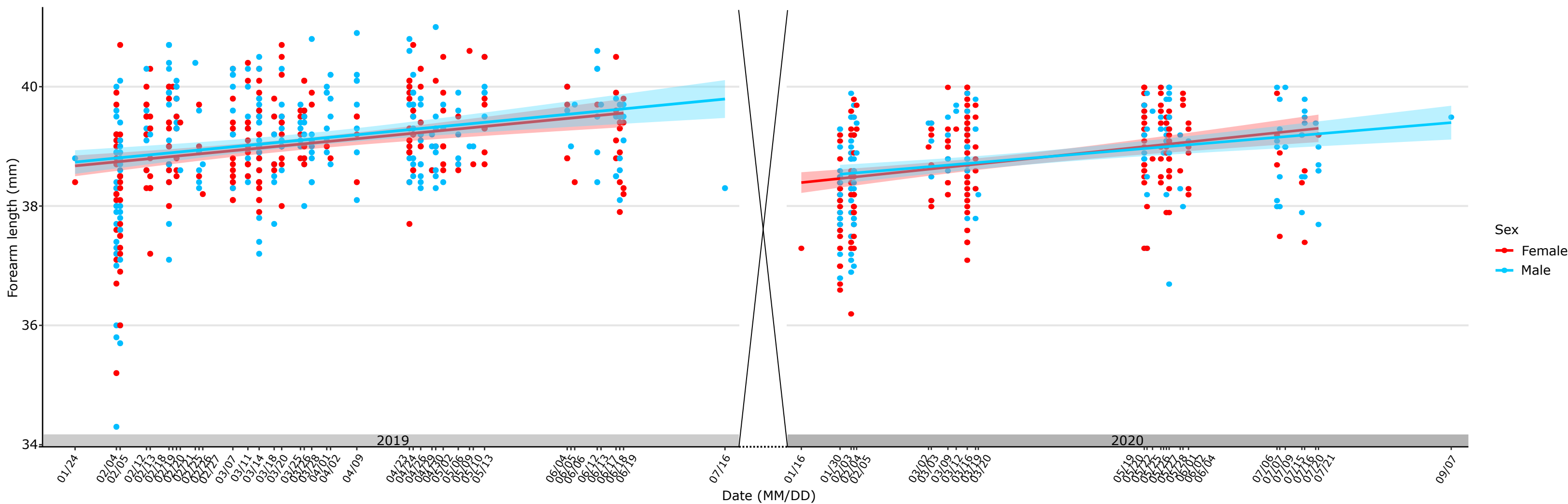

Supplement: Supplementary file 6 — Figure S6 [file ECE3-13-e9814-s008.pdf]

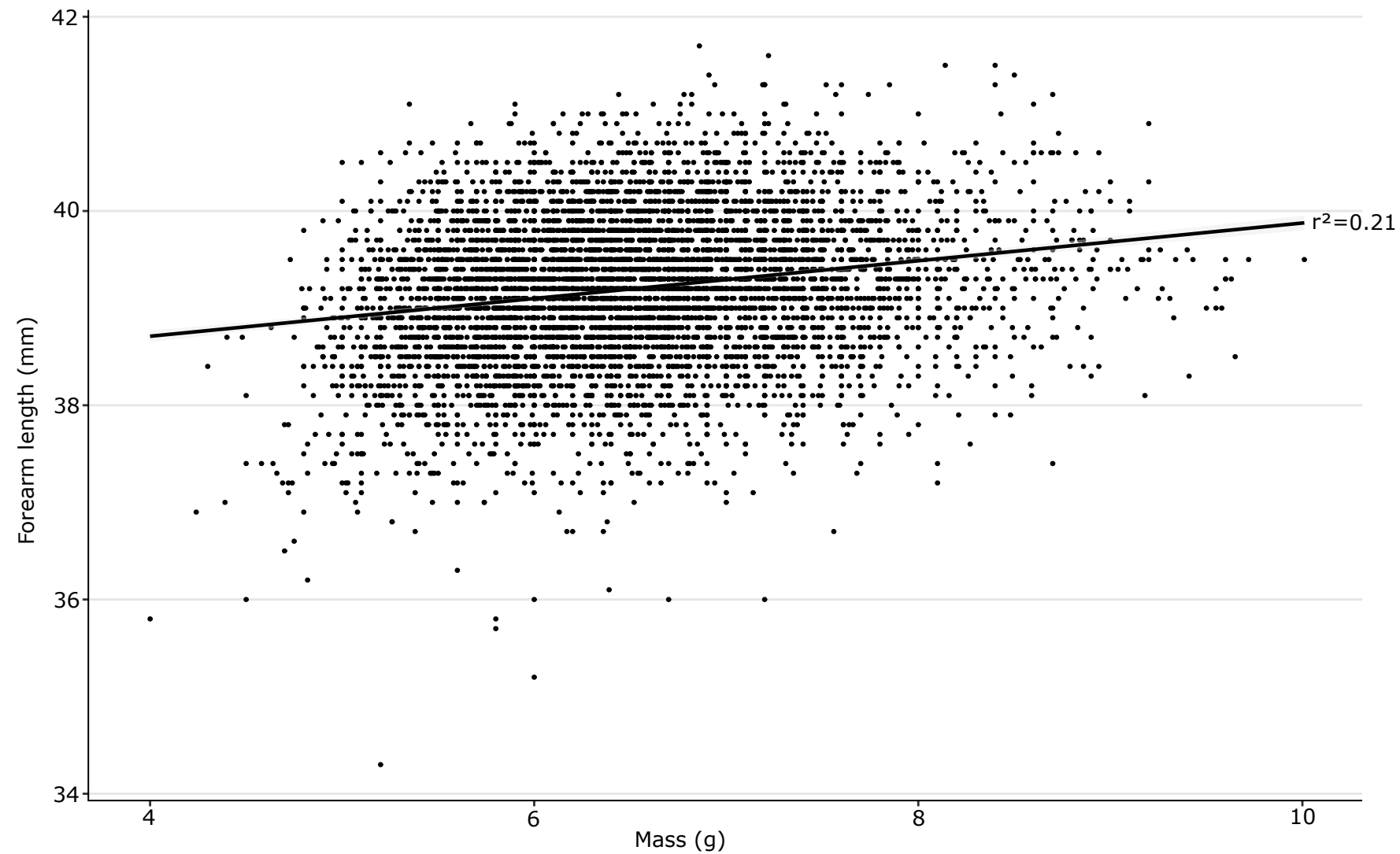

Supplement: Supplementary file 7 — Figure S7 [file ECE3-13-e9814-s006.pdf]

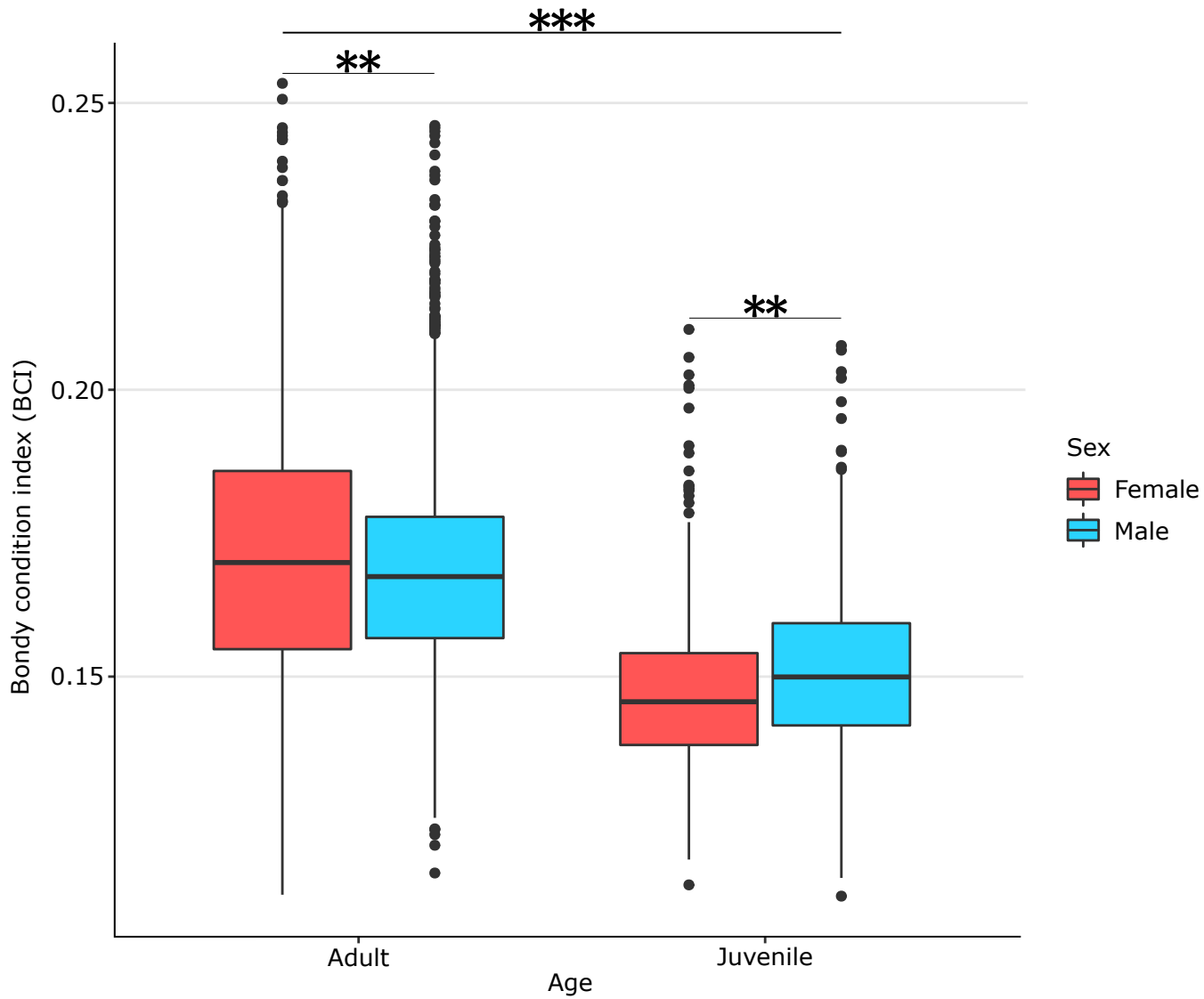

Supplement: Supplementary file 8 — Figure S8 [file ECE3-13-e9814-s010.pdf]
