# Supplementary material for: A population in perpetual motion: Highly dynamic roosting behavior of a tropical island endemic bat
Source: Ecol Evol. 2023 Feb 11;13(2):e9814. doi: 10.1002/ece3.9814 (PMC9919472; doi:10.1002/ece3.9814)
Supplement: Supplementary file 9 — Table S1 [file ECE3-13-e9814-s003.pdf]

Year	Sampling period	Capture dates (MM/DD) (number of captured individuals)
2018	November - December	From 10/26 to 12/07 (n = 601)
	December	From 12/10 to 12/17 (n = 164)
2019	January - February	From 01/24 to 02/27 (n = 555)
	March - April	From 03/07 to 04/09 (n = 534)
	April - May	From 04/23 to 05/13 (n = 478)
	June	From 06/04 to 06/19 (n = 317)
	July	From 07/16 to 07/30 (n = 229)
	September	From 09/03 to 09/17 (n = 325)
	October - November	From 10/21 to 11/07 (n = 510)
	November - December	From 11/25 to 12/10 (n = 379)
2020	January - February	From 01/15 to 02/05 (n = 525)
	March	From 03/02 to 03/20 (n = 413)
	May - June	From 05/19 to 06/04 (n = 331)
	July	From 07/06 to 07/21 (n = 190)
	September	From 09/07 to 09/24 (n = 310)
	October	From 10/14 to 10/29 (n = 453)
	November - December	From 11/23 to 12/09 (n = 407)
