# Supplementary material for: A population in perpetual motion: Highly dynamic roosting behavior of a tropical island endemic bat
Source: Ecol Evol. 2023 Feb 11;13(2):e9814. doi: 10.1002/ece3.9814 (PMC9919472; doi:10.1002/ece3.9814)
Supplement: Supplementary file 10 — Table S2 [file ECE3-13-e9814-s009.pdf]

Scale of analysis	Category of individuals included	N	Model number	Complete model	Selected model
1) <u>Roost size</u>					
Log (Roost Size)	All individuals	6,721	$GAMM_1$	Period + (Roost) + (EstimSizeMethod)	Period + (Roost)
2) <u>Sex-ratio</u>					
Sex-ratio	All individuals	6,721	LMM_2	Period + (Roost)	Period + (Roost)
3) <u>Sexual dimorphism</u>					
Forearm length	Adults<br>(excluding data from recaptures)	5,241	LMM_3	Sex + (Roost)	Sex + (Roost)
Forearm length	Juveniles<br>(excluding data from recaptures)	953	LMM_4	Sex + Date + Sex : Date + (Roost) + (Year)	Sex + Date + (Roost) + (Year)
4) <u>Body condition</u>					
BCI	All individuals	6,721	LMM_5	Sex + Age + Sex : Age + (Period) + (Roost)	Sex + Age + Sex : Age + (Period) + (Roost)
BCI	Adult females	2,471	LMM_6	Repro + (Period) + (Roost)	Repro + (Period) + (Roost)
BCI	Adult males	3,292	LMM_7	Repro + (Period) + (Roost)	Repro + (Period) + (Roost)
BCI	Adult females<br>(Gestation period)	584	LMM_8	logSize + Repro + logSize : Repro + (Year/Date) + (Roost)	Repro + logSize : Repro + (Year/Date)
BCI	Adult females<br>(Gestation period) without TBA	498	LMM_9	logSize + Repro + logSize : Repro + (Year/Date) + (Roost)	Repro + (Year/Date)
BCI	Adult males<br>(Mating period)	294	LMM_{10}	logSize + Repro + logSize : Repro + (Year/Date) + (Roost)	Repro + (Year/Date)
